# Feeding, Muscle and Packaging Effects on Meat Quality and Consumer Acceptability of Avileña-Negra Ibérica Beef

**DOI:** 10.3390/foods9070853

**Published:** 2020-06-30

**Authors:** Marta Barahona, Mohamed Amine Hachemi, José Luis Olleta, María del Mar González, María del Mar Campo

**Affiliations:** 1 Department of Animal Production and Food Science, Instituto Agroalimentario (IA2), Universidad de Zaragoza-CITA, Miguel Servet 177, 50013 Zaragoza, Spain; hachemi.inmv@gmail.com (M.A.H.); olleta@unizar.es (J.L.O.); marimar@unizar.es (M.d.M.C.); 2 Asociación Española de Raza Avileña-Negra Ibérica, Padre Tenaguillo 8, 05004 Ávila, Spain; consejoregulador@carnedeavila.org

**Keywords:** concentrate, silage, modified atmosphere, vacuum, texture, fatty acids, water holding capacity, consumer acceptability

## Abstract

In order to achieve an attractive and differentiated product for the consumer and to optimize and to maximize profitability for the farmers within the EU Protected Geographical Indication “Carne de Ávila”, 24 yearling males of Avileña-Negra Ibérica breed were used to evaluate the effect of 2 feeding systems, concentrate (CON) and maize silage (SIL), and 2 packaging systems, vacuum (VAC) and modified atmosphere (MAP), on fatty acid composition, proximate analysis, water holding capacity and consumer acceptability of meat in 2 muscles: *Longissimus thoracis* (LT) and *Semitendinosus* (ST). Animals fed with concentrate showed higher carcass weight. However, feeding did not affect the proximate analysis of the meat. The use of maize silage improved the amount of conjugated linoleic acid and *n*-3 polyunsaturated fatty acids (PUFA) and the relation *n*-6 PUFA/*n*-3 PUFA. In LT muscle, feeding influenced texture, samples from SIL being more tender. The VAC packaging showed higher cooking losses than MAP in both muscles. Aging increased tenderness and cooking losses but decreased thawing losses. LT samples from SIL feeding were better accepted by consumers and VAC packaging showed higher scores than MAP. We can conclude that the use of maize silage could be an alternative feeding for this type of animals improving some aspects of the quality of the meat.

## 1. Introduction

During the past decades, consumers have shown an increasing interest in food production including animal origin [1] at the same time that beef consumption has decreased. Besides the increasing numbers of vegetarians and vegans [2,3], several factors related to the quality of the product have motivated this negative tendency such as animal health [4], origin [5,6], genetic factors [7,8], feeding systems [9,10,11,12], conditioning and processing of the meat [13], among others.

The European Union includes within the label Protected Geographical Indication (PGI) those products that are reared in traditional or well-defined production systems, which in the end promotes a clear diversification of agricultural production and specific products [14]. In that sense, the Avileña-Negra Ibérica local beef breed (*Bos taurus*) is traditionally produced in the western region of Spain and since 1996 shows PGI label [15]. After weaning, calves are generally fattened in confinement and fed with cereal straw and concentrates, until they reach around 12–14 months of age with approximately 500 kg of live weight (550 kg for entire males and 450 kg for females), obtaining a meat that improves its quality with aging [16].

The producers of beef in several countries have had an important crisis of profitability in recent years due to the high price of some raw materials for feeding their animals and the high purchase cost of the calves. More extensive production systems, with pasture or forage, have lowered costs compared with diets based on concentrates. However, the agro-climatic conditions of an area or the farm structure do not always allow rearing efficiently the animals in those systems. Several studies have analysed carcass and meat quality of this local breed alone or compared with others [16,17,18,19,20]. The forages are cheaper than concentrates and allow an integration of the animal into sustainable rural environments; moreover, they are rich in natural antioxidants, plant pigments and *n*-3 polyunsaturated fatty acids if they are composed of pasture grasses and legumes [21]. However, feeding diets based on silage are a feasible option because they can easily be produced in irrigated areas located near the feedlots where grazing is not an option. Including maize silage in diets given as total mixed rations for fattening cattle has aroused much interest in recent years [22,23,24,25]. Maize silage lowers the cost of rations by increasing forage consumption without decreasing energy concentration, while the risk of low roughage supply due to improper processing in the mixer wagon can easily be avoided by adding small amounts of grass hay or cereal straw to the diet [26].

On the other hand, there is a gradual appearance of brands that want to guarantee the quality of the product: Quality and safety can be the key to the future. Consumers have evolved in their criteria with respect to food selection. The type of packaging used will influence in the decision to choose meat from a refrigerated display cabinet at purchase [27]. Packaging systems vary in the equipment required, the visual appeal to consumers, shelf life and effects on eating quality. There is a tendency to select packaged meat with modified atmospheres, because the high content of oxygen produces a red brilliant colour in the surface of the meat, which is generally considered desirable by consumers.

Thus, the objectives of this work were to study the effect of the production system (concentrate vs. maize silage) and packaged method (MAP and vacuum) on instrumental beef quality and acceptability of Avileña-Negra Ibérica breed within Carne de Ávila IGP label, in two muscles (*Longissimus thoracis* and *Semitendinosus*). In this sense, the knowledge of the relationships among production, meat quality, instrumental and sensory quality could help improving the final product, as well as understanding consumer acceptability.

## 2. Materials and Methods

### 2.1. Experimental Design and Animal Management

This study was performed with animals reared in a farm under commercial practices following national regulations in animal welfare (Council Directive 2008/119/EC), with animals being slaughtered at a commercial abattoir with captive bolt stunning in agreement with regulation 1099/2009 of the European Union about protection of animals at the time of killing.

The experimental treatments were conducted with yearlings from Avileña-Negra Ibérica breed in Spain. Twenty-four young bulls (250.7 kg ± 64.7 standard deviation initial live weight and 200.5 days old ± 44.9 standard deviation) were selected randomly from commercial indoor batches of 200 animals and controlled through the trial. Groups of four bulls were reared in separate pens for a total of 3 pens per group with 4.8 m^2^ space allowance per animal. These were assigned to two groups: One group of 12 animals (CON) was fed with concentrates and cereal straw ad libitum, coincidentally with the most common husbandry conditions in the country for intensive rearing, and the other group of 12 animals (SIL) was fed with a mixture of 70% of maize silage and 30% of concentrate also ad libitum using a unifeed mixer wagon. The raw components of the CON group diets were barley, corn, dried waste from corn distillery, rapeseed flour, soybean flour, palm oil, calcium carbonate, palm fatty acids salt, sodium carbonate, salt and magnesium oxide. In the case of the SIL group, the ingredients used were similar with the exception of barley that was not included, and the rapeseed flour was replaced by shelled soybeans. Water was administrated ad libitum in both groups and days in trial were 276 in CON and 233 in SIL.

The composition of these diets per 100 g was CON: 379 kcal of calorific value, 8.03 g of moisture, 6.73 g of ash, 7.88 g of fat, 14.6 g of crude protein, 70.8 g of total carbohydrates and SIL: 205 kcal of calorific value, 50.7% moisture, 5.51 g of ash, 5.90 g of fat, 12.3 g of crude protein, 76.2 g of total carbohydrates. Fatty acid composition (g/100 g of total fat) is presented in Table 1.

### 2.2. Slaughter and Meat Sampling

The target for slaughter was visual fatness for this breed under commercial practices, performed at the farm by an expert, reaching a minimum of 3.5 on a 5-point-scale [28]. After a finishing period of approximately of 250 days, the bulls with the average weight of 578.2 kg ± 36.4 standard deviation kg and 455.2 days old ± 41.6 standard deviation the animals were slaughtered in an EU licensed abattoir, and carcass conformation and fatness were obtained according to EU regulations [29]. Carcasses were chilled at 4 °C under commercial conditions. At 72 h post-mortem, *Longissimus thoracis* (LT) and *Semitendinosus* (ST) muscles were removed from the left side of each carcass and immediately vacuum packaged. They were stored and transported under refrigeration at 4 °C to the Veterinary Faculty of the University of Zaragoza.

At 7 days post-mortem, steaks from each animal were obtained for the analysis of fatty acids, texture, water holding capacity and consumer’s test.

### 2.3. Proximate Composition and Fatty Acids

Steaks (1 cm thick) for chemical composition were removed between T6 and T7 from LT and from the distal side of ST. The samples were packaged under vacuum conditions and immediately frozen at −18 °C for posterior analysis. The composition of moisture [30], ash [31], lipid [32] and protein [33] was analysed according to official methods. For the fatty acid analysis, intramuscular fat was extracted in chloroform: methanol [34]. The methyl ester preparation included KOH in methanol, with C19:0 as an internal standard and was analysed by gas chromatography in HP 6890 equipped with an ionization flame and an automatic injection system (HP 7683) and fitted with a SP 2380 column (100 m × 0.25 mm × 0.20 µm) and oven temperature programming as follows: column temperature was set at 140 °C, then raised at rate of 3 °C/min from 140 °C to 158 °C, and 1 °C /min to 165 °C, kept for 10 min, raised at 5 °C /min up to 220 °C and kept constant for 50 min. Inlet temperature was kept at 230 °C and detector at 240 °C. A split mode injector with split ratio of 1/32 was applied; nitrogen as the carrier gas was used at a constant flow rate of 0.8 mL/min with an injected volume of 1 µL; methyl esters were identified using retention times of Sigma chemical Co; Standards [35]. Each sample was analysed in duplicate, and results were expressed as a percentage of total fatty acids.

### 2.4. Texture Analysis

Texture was measured at 7, 14 and 21 days of aging. Steaks with 7 days of aging from both muscles were vacuum packaged and immediately frozen at −18 °C. Half of the steaks for 14 and 21 days of aging were vacuum packaged and kept in the dark in the refrigerator to avoid oxidation. The other half were packaged with modified atmosphere (MAP) with 70% O_2_:30% CO_2_, remaining displayed at 4 °C and light (1200 luxes for 12 h/day) in a commercial cabinet with doors to promote oxidation, in conditions that are also commercially used. When reaching 14 or 21 days of aging, the steaks in MAP were repackaged in vacuum bags, frozen and kept at −18 °C until analysed.

Samples were thawed 24 h before the analysis at 4 °C in batches of 12 steaks selected randomly. Texture analysis was performed using two methods: Warner Bratzler Shear Force (WBSF) in cooked meat and compression in raw meat. In the analysis of WBSF, the steaks were cooked under vacuum in a water bath at 75 °C, until reaching an internal temperature of 70 °C, and then cooled in cold water. After cooling, samples were cut in the direction of the muscle fibres, and an average of six parallelepiped samples of 1 cm^2^ cross section were obtained and assessed with an Instron Texturometer 4301 equipped with a WBSF cell. Samples were placed so that the muscle fibre direction was perpendicular to the load cell. Values obtained were the shear force (kg) and toughness (kg/cm^2^), considered as the energy required cutting the sample at the point of maximum stress), collecting the average of a minimum of four replicates per each sample. Texture of raw meat was analysed using a modified compression device that hinders transversal elongation of the sample [36], also with an average of six parallelepipeds samples of 1 cm^2^ cross section. Values were recorded at 20% of compression rate (C20; N/cm^2^), which is related to the strength of the muscle fibre, and 80% of compression rate (C80; N/cm^2^), which is related to the strength of the connective tissue.

### 2.5. Water Holding Capacity (WHC)

Steaks intended for texture analysis were used to measure the WHC as water losses during conservation. Water losses were calculated by the difference in weights measured before and after exposure of the steaks during 14 and 21 days of aging (7 and 14 days of conservation respectively) at vacuum and MAP. Water losses during thawing and during cooking were calculated from the differences in weights previously obtained and subsequently thawing and cooking steaks 7, 14 and 21 days of ripening MAP and vacuum packed. The WHC is calculated according to the equation: WHC (%) = [(initial weight − weight final)/initial weight] × 100.

### 2.6. Consumer Analysis

The samples for consumer test of both muscles (LT and ST) were packaged in MAP and vacuum and remained on display in a refrigerator at 4 °C in light (those packaged in MAP) and in the darkness (those packaged in vacuum) until reaching 14 days of aging. Then, all of them were vacuum packaged and frozen at −18 °C until the day of analysis.

Samples were thawed and kept at 4 °C and darkness 24 h before the analysis. For cooking, steaks were kept at room temperature for one hour before they were placed in aluminium foil, coded with three-digit number assigned randomly and cooked in an industrial double plate grill SAMMIC GRS-5, pre-heated at 200 °C, without adding any salt or spices, until reaching an internal temperature of 70 °C controlled by internal thermocouple (JENWAY, 2000. Staffordshire, UK).

From each cooked sample and after removing the external fat, 10 pieces of equal size (2 × 2 × 2 cm) were obtained. They were wrapped in aluminium foil previously labelled with a three-digit number and maintained at 50 °C until presented to the consumers, delaying less than 10 min.

A total of 120 consumers participated in the analysis. Each consumer received eight samples in different order at random inside each muscle, so that the first four samples were from the LT, followed by four from the ST. The acceptability of tenderness, flavour and overall appraisal on a scale of nine points were assessed from 1 (I dislike extremely) to 9 (I like extremely). The midpoint of the scale was removed to force a positive or negative decision by the consumer [37].

### 2.7. Statistical Analysis

All data were analysed using the SPSS 22.0 statistical package. The analysis of fatty acids and proximate analysis were analysed by the GLM procedure considering feeding and muscle as fixed effects. Texture measurements, water holding capacity and consumers test were divided by muscle and analysed with the GLM procedure considering feeding, packaging and aging as fixed effects and covariance by carcass weight. In the consumer test, consumer was considered a random effect and carcass weight as a covariate. The Duncan’s multiple range test with a significance of *p <* 0.05 was used to assess differences between average values

## 3. Results and Discussion

### 3.1. Production Traits and Carcass Quality

Animals were introduced in the experimental phase with a similar age and a small difference in live weight (*p* < 0.1), which showed a tendency to be bigger in the maize silage group than in the one with concentrate (273.83 kg vs. 227.67 kg). However, slaughter age, slaughter live weight and carcass weight differed significantly between lots, with higher average values in CON group than in SIL group: 473.17 days, 595.67 kg and 341.80 kg vs. 437.17 days, 560.67 kg and 318.37 kg, respectively (Table 2). This was probably due to the chosen target for slaughter, which was visual fatness before slaughtering and was reflected in there being no differences in carcass conformation or fatness. These two characteristics are essential for assessing the final price of the carcass [29].

No differences were found in average daily gain (ADG), similar in both groups. The same results were obtained by Casasús et al. (2012) [25] in a study comparing the use of unifeed with 80% maize silage and 20% concentrate and Avilés et al. (2015) [20] with a diet unifeed composed of the same proportion of concentrate, maize silage and wheat straw. On the contrary, other authors have found that the ADG of animals fed with concentrate was bigger than in those fed with a base of grass silage [38]. Furthermore, Steen et al. [39] found a higher ADG (38%) in animals receiving grass silage and concentrate that in the animals fed only with grass silage, because of the higher energy content of the concentrate. This might have also happened in our experiment; although ADG was not significantly different between treatments, CON animals were longer in the trial and reached a significantly heavier weight at slaughter (Table 2), with a diet higher in energy.

The ADG is similar to that found by Campo et al. [16] for this breed but much lower than that published by Piedrafita et al. [40] with 1.64 kg/d. The slaughter age in our study was a bit older than the slaughter age in the latter case, which was between 12 and 13 months, and this could explain these differences. When slaughtering animals older than 14 months, the growth in the last stage is based in fat deposition and, therefore, the weight increase slows down. However, in our study, there were not significant differences in carcass fat deposition (Table 1). The conformation tended to be slightly higher in animals fed with concentrates than in animals with maize silage, probably as a result of the higher carcass weight. Similar results observed Realini et al. [21] in a study comparing animals finished with grass with animals finished with silage. Moreover, Casasús et al. [25] observed better conformation in animals fed with concentrates, again due to the higher energy in the feeding.

### 3.2. Proximate Analysis

Diet did not have an effect on proximate composition (Table 3), but the type of muscle had a significant effect in the percentage of ashes. Previous studies with the same breed showed higher intramuscular fat in meat from animals fed under free-range conditions and supplemented with concentrate than in meat from those finished in confinement with cereal straw and concentrate [15]. The target of similar fatness at slaughter might be the responsible for this lack of differences in our study. The amount of intramuscular fat in beef from Avileña-Negra Ibérica breed was around 4%. This value would be classified as lean meat according to Food Advisory committee (1990) [41]. However, other authors found lower values in studies carried out with this same breed [16,40].

Some authors [42] have reported a linear relationship between fat and protein content. Acheson et al. [42] reported that ash values increase with fat as quality grade increases, and higher quality grade is positively correlated with higher intramuscular content. Although the ash content was significantly higher in ST than in LT (1.24% vs. 1.10%, respectively), we have not found this relationship with protein or fat content, since no statistical differences were found.

### 3.3. Fatty Acids

The fatty acid composition of the muscles and diets is presented in Table 4. LT showed higher percentage of saturated fatty acids than ST. These differences were mainly due to the amounts of the more common saturated fatty acids (SFA) presented in the meat, such as myristic acid (C14:0), palmitic acid (C16:0) and stearic acid (C18:0) at the cost of polyunsaturated fatty acids (PUFA). There were no differences in monounsaturated fatty acids (MUFA) between muscles. However, the percentages of *t*C18:1*n*-10+11 and C17:1 were significantly higher in LT than in ST. Oleic acid (C18:1-*n*-9) was similar in both muscles and C18:1*n*-11 was higher in ST than LT muscle.

The effect of muscle was significant for all the PUFA, with higher concentration in ST than in LT. The linoleic acid was the predominant PUFA presented in the muscle and significantly higher in ST than LT (13.65% vs. 9.31%, respectively). PUFA are predominant in “red” muscles, such as ST, due to the higher content of phospholipids in them in relation to white muscles [43]. Although LT is not a strictly “white” muscle because of the mix of fibres that it has, it is different enough from ST in its fibre composition.

The effect of the diet on fatty acid composition was significant for the total SFA and MUFA. The use of maize silage produced meat with higher percentage of *n* − 3 PUFA. However, the percentage of *n* − 6 PUFA was not affected by the diet, although SIL showed high content of 18:2 *n* − 6 in the feed. The variability between animals are responsible for the no differences between the average values of intramuscular fat (4.5% vs. 3.6%). A higher saturation of meat from animals fed with SIL could be due to the higher incorporation of fat in the neutral lipids fraction because in ruminant muscle most PUFA are located in the phospholipid fraction [43]. Other authors have reported similar results. Warren et al. [44] observed that the use of ray-grass silage decreased the relationship PUFA/SFA, increasing the saturation. That increase was because of the reduction of MUFA, especially the percentage of C18:1 *n* − 9 that was lower in animals fed SIL than in CON animals (29.32% vs. 32.82%, respectively) as a reflection of the lower content in the SIL diet vs. CON. Casasús et al. [25] also found a decrease of ratio *n* − 6/*n* − 3 because of the higher proportion of the *n* − 3 PUFA in the meat of animals fed with maize silage. The higher proportion of *n* − 3 PUFA is associated to the composition of the silage, different to the concentrate, because the cereals and soya had higher content of *n* − 6 PUFA than silage. Some authors have showed that the use of maize silage could cause an increase of SFA and PUFA at the same time that decreases the MUFA [45,46]. As a result of the higher composition of *n* − 3 PUFA, the ratio *n* − 6 PUFA/*n* − 3 PUFA was lower (*p* < 0.001) in SIL than in CON animals (11.5 vs. 15.4). These values are typical in intensively fed animals that do not graze [43] but are far from the recommended level below 4 in the diet in terms of human health [47]. The ratio PUFA/SFA, which should be over 0.4 in a healthy diet [47], was reached by both groups without significant differences between them.

### 3.4. Texture Analysis and Water Holding Capacity

There was an effect of the feed on maximum load and toughness in LT (Table 5). Meat from CON group was tougher than meat from SIL group. The meat texture is very important for satisfying the parameters required by consumers, especially in beef where this organoleptic characteristic is valued primarily for consumption [48]. Even more, Hoving-Bolink et al. [49] found higher meat tenderness in animals reared with maize silage than in those fed with grass silage. Texture, considered as WBSF, has a high variability, which is attributable to factors related to animals, environment, pre-slaughter or breed [16], sex [50], age at slaughter [51], slaughter weight [52], feeding system, feeding level [53], compensatory growth [54], average daily gain, physical activity or confinement time [55] as well as technological factors, maturation, packaging, temperature and cooking techniques.

Another indicator associated with meat tenderness is the water losses, where meats having greater water loss usually have lower tenderness [56]. Still, the water holding capacity decreases with the increase in age of the animal [57]. Some authors have found differences between breeds [58], and the breeds with larger average daily gain presented lower WHC.

As it is shown in Table 4, in the muscle LT, thawing losses were affected by packaging and aging. Samples packaged in MAP and aged for 7 days showed the higher thawing losses. However, water losses were higher in samples from VAC and aged for 21 days. Cooking losses were affected by feeding in muscle ST (Table 6), where the samples from CON showed higher losses than samples from SIL feeding. Thawing and display losses were affected by packaging and aging; the same as occurred in muscle LT.

The increase in water losses during display (LT and ST) over time may be a consequence of the proteolysis that takes places during tenderization [59], which decreases the retention force of water by structural proteins. Display losses were greater in meat in vacuum than in MAP, and this could be due to the negative pressure the muscle receives during vacuum, which could facilitate the loss of part of the exudate.

The least thawing loss in the most exposed meat could be because some of the water was lost during display prior to be frozen. The losses were similar for all three aging times, with a trend of increased average losses in meat from animals fed with maize silage when compared to the concentrate fed. On the other hand, in the ST, the type of feeding did not affect any type of water losses analysed.

### 3.5. Consumer Analysis

Consumers’ scores are presented in Table 7 and Table 8. The results were separated by muscle. In the case of LT, the effect of the feed was significant for global and tenderness acceptability, being samples from SIL better accepted than CON. For flavour acceptability, feeding had no effect on consumer’s perception. However, those differences were not appreciated in the muscle ST, where feeding had no effect on consumer acceptability. Other authors had also reported this lack of effect of the feeding [15,20,60].

In addition, greater acceptability notes of tenderness and flavour for LT when compared to the ST were observed. Moreover, the samples packaged in vacuum had higher global notes when comparing with the samples packaged in the MAP. MAP is rich in oxygen to improve and keep an attractive colour during display, but it favours oxidation. Oxidation provokes more rancid notes during conservation [61], and this can reduce flavour acceptability after consumption.

Feeding affected the acceptability of LT, where consumers preferred meat from SIL group. However, those differences only appeared for global acceptability and tenderness but not flavour. Tenderness is one of the sensory quality parameters most affected by aging since it increases tenderness due to proteolysis, and most consumers prefer tender meat [16]. On the other hand, sometimes meat packaged in MAP has been found less tender than vacuum packaged meat [62] similar to the findings of this study. This effect in high oxygen packaging could be explained by the decrease in the proteolysis of myofibrils, because of the decrease in the µ calpain activity [63]. Consumers found significant differences for packaging. In both muscles, the scores for the three attributes were higher than 6 in samples from vacuum packaging in comparison with MAP packaging, especially in muscle LT, in which the scores were higher than 6.5 in a 9 points scale.

## 4. Conclusions

The use of maize silage as an alternative to conventional concentrate during the confinement period did not alter the productive parameters in terms of average daily gain (ADG), carcass percentage and fatness score. Maize silage would be recommended for improving tenderness of Avileña-Negra ibérica beef, especially in the case of muscles with higher commercial category such as LT. However, aging of the meat it is still one of the best ways to improve its tenderness.

Regarding the sensory quality, vacuum aging improves overall, tenderness and flavour acceptability; moreover, the LT muscle shows a greater global and flavour acceptability when compared to the ST. Therefore, confinement beef with maize silage may be an alternative that meets the quality requirements of the market and of consumers of beef.

## Figures and Tables

**Table 1:** Fatty acids (g/100 g of total fat) of the diets (CON: concentrate; SIL: 70% maize silage + 30% concentrate).

	CON	SIL
**C6:0**	-	0.04
**C8:0**	-	0.02
**C12:0**	0.13	0.11
**C14:0**	0.44	0.27
**C16:0**	24.14	16.59
**C16:1**	0.14	0.13
**C17:0**	-	0.07
**C17:1**	-	0.03
**C18:0**	2.81	2.35
**C18:1**	31.91	29.27
**C18:2**	38.00	46.51
**C18:3**	1.43	1.79
**C20:0**	0.43	0.61
**C20:1**	0.29	0.23
**C22:0**	-	0.15

**Table 2:** Production traits and meat quality of Avileña-Negra Ibérica yearlings feeding with concentrate (CON) or 70% maize silage + 30% concentrate (SIL).

	CON	SIL	SEM	Significance
***n***	*12*	*12*		
**Initial age d**	196.8	204.2	9.36	0.696
**Initial weight kg**	227.7	273.8	12.58	0.080
**Slaughter age d**	473.2 a	437.2 b	7.79	0.031
**Slaughter weight kg**	595.7 a	560.7 b	6.62	0.015
**Carcass Weight kg**	341.8 a	318.4 b	3.82	0.006
**ADG kg/d**	1.34	1.26	0.04	0.298
**Conformation**	8.58 (R+)	7.83 (R)	0.206	0.083
**Fatness**	8.00 (3)	7.42(2+)	0.179	0.117
**Carcass yield %**	57.4	56.8	0.287	0.304

SEM: Standard error mean. a, b: different letters indicate significant differences in the mean values (*p* < 0.05).

**Table 3:** Effect of feeding (concentrated (CON) and 70% maize silage + 30% concentrate (SIL)) and muscle (*Longissimus thoracis* (LT) and *Semitendinosus* (ST)) on proximate analysis of meat from Avileña-Negra Ibérica breed.

	FEEDING		MUSCLE		Significance			
	SIL	CON	LT	ST	SEM	FEED	MUS	FEED × MUS
***n***	*24*	*24*	*24*	*24*				
**Moisture**	72.5	73.0	72.5	72.9	0.207	0.401	0.499	0.833
**Protein**	21.9	22.2	22.0	22.1	0.208	0.409	0,802	0.802
**Lipid**	4.47	3.63	4.36	3.73	0.277	0.138	0.261	0.766
**Ashes**	1.16	1.18	1.10 b	1.24 a	0.014	0.710	<0.001	0.113

SEM: standard error mean; a, b: different letters indicate significant differences in the mean values (*p* < 0.05) within effects.

**Table 4:** Effect of muscle *Longissimus thoracis* (LT) and *Semitendinosus* (ST) and feeding (concentrate (CON) and 70% maize silage + 30% concentrate (SIL)) on fatty acids composition (g/100 g of total fatty acids) of meat from Avileña-Negra Ibérica breed.

	LT	ST	CON	SIL	SEM	Muscle	Feed	Feed × Mus
***n***	*24*	*24*	*24*	*24*				
**C10:0**	0.03 a	0.02 b	0.03	0.03	0.002	0.001	0.680	0.037
**C12:0**	0.04 a	0.03 b	0.04	0.03	0.002	0.031	0.071	0.558
**C13:0**	0.01 a	0.00 b	0.01	0.01	0.000	0.001	0.042	0.719
**C14:0**	2.09 a	1.59 b	1.91	1.77	0.070	<0.001	0.226	0.230
**C15:0**	0.27	0.24	0.28 a	0.23 b	0.006	0.049	<0.001	0.353
**C16:0**	24.39 a	22.54 b	23.11	23.83	0.264	<0.001	0.109	0.062
**C17:0**	0.85 a	0.76 b	0.86 a	0.74 b	0.017	0.003	<0.001	0.626
**C18:0**	18.10 a	13.44 b	14.96 b	16.58 a	0.496	<0.001	0.026	0.455
**C19:0**	0.17	0.16	0.16	0.17	0.004	0.194	0.157	0.829
**C20:0**	0.12 a	0.09 b	0.11	0.11	0.004	<0.001	0.526	0.869
**C21:0**	0.28	0.32	0.27 b	0.33 a	0.012	0.106	0.013	0.135
**C22:0**	0.05 b	0.08 a	0.04 b	0.09 a	0.019	<0.001	<0.001	0.001
**C14:1**	0.25	0.27	0.31 a	0.21 b	0.001	0.508	0.015	0.518
**C15:1**	0.01	0.01	0.01	0.01	0.001	0.563	0.026	0.626
**C16:1**	2.02	2.15	2.36 a	1.82 b	0.084	0.387	0.001	0.515
**C17:1**	0.45	0.52	0.58 a	0.40 b	0.087	0.043	<0.001	0.885
**tC18:1*n* − 10 + 11**	2.74 a	2.27 b	2.48	2.53	0.087	0.006	0.783	0.470
**C18:1*n* − 9**	31.37	30.77	32.82 a	29.32 b	0.529	0.535	0.001	0.941
**C18:1*n* − 11**	1.33 b	1.53 a	1.57 a	1.28 b	0.035	<0.001	<0.001	0.506
**C18:1*n* − 13**	0.24	0.24	0.20 b	0.28 a	0.018	0.884	0.023	0.703
**C20:1**	0.13	0.15	0.14	0.14	0.005	0.140	0.531	0.201
**C22:1*n* − 9**	0.04	0.04	0.06 a	0.02 b	0.008	0.597	0.025	0.048
**tC18:2*n* − 6**	0.13 b	0.16 a	0.13 b	0.16 a	0.005	0.005	0.017	0.401
**C18:2*n* − 6**	9.31 b	13.65 a	11.22	11.74	0.521	<0.001	0.544	0.293
**C20:2*n* − 6**	0.06 b	0.10 a	0.07 b	0.09 a	0.004	<0.001	<0.001	0.004
**C20:2*n* − 3**	0.07 b	0.14 a	0.10	0.12	0.008	<0.001	0.075	0.091
**C22:2*n* − 6**	0.01 b	0.02 a	0.01 b	0.02 a	0.001	<0.001	<0.001	0.066
**C18:3*n* − 6**	0.05 b	0.07 a	0.05 b	0.07 a	0.003	0.001	<0.001	0.020
**C18:3*n* − 3**	0.25 b	0.34 a	0.25 b	0.34 a	0.013	<0.001	<0.001	0.025
**C20:3*n* − 6**	0.33 b	0.60 a	0.37 b	0.56 a	0.033	<0.001	<0.001	0.014
**C20:3*n* − 3**	0.07	0.11	0.04 b	0.14 a	0.012	0.041	<0.001	0.726
**C20:4*n* − 6**	1.98 b	3.67 a	2.57	3.08	0.185	<0.001	0.064	0.175
**C20:5*n* − 3**	0.12 b	0.27 a	0.17	0.22	0.018	<0.001	0.087	0.059
**C22:5*n* − 3**	0.29 b	0.69 a	0.39 b	0.58 a	0.042	<0.001	0.001	0.027
**C22:6*n* − 3**	0.02 b	0.06 a	0.03 b	0.05 a	0.003	<0.001	0.009	0.135
**SFA**	46.47 a	39.63 b	41.91 b	44.18 a	0.638	<0.001	0.003	0.055
**MUFA**	38.57	37.94	40.51 a	35.99 b	0.639	0.581	<0.001	0.927
**PUFA**	12.98 b	20.21 a	15.68	17.51	0.806	<0.001	0.137	0.186
***n* − 6**	11.88 b	18.26 a	14.41	15.73	0.729	<0.001	0.249	0.212
***n* − 3**	0.82 b	1.62 a	0.99 b	1.45 a	0.089	<0.001	<0.001	0.035
**PUFA/SFA**	0.28 b	0.52 a	0.38	0.42	0.024	<0.001	0.318	0.135
***n* − 6/*n* − 3**	14.82 a	12.08 b	15.37 a	11.53 b	0.464	<0.001	<0.001	0.358

SEM: standard error mean; a, b: different letters indicate significant differences in the mean values within effects (*p* < 0.05). SFA: Saturated fatty acids; MUFA: Monounsaturated fatty acids; PUFA: Polyunsaturated fatty acids.

**Table 5:** Effect of feeding (concentrate (CON) vs. 70% maize silage + 30% concentrate (SIL)) and packaging (vacuum (VAC) and modified atmosphere (MAP)) and aging (7, 14 and 21 days) on texture parameters (maximum load and toughness) water holding capacity (WHC) of thawing, cooking and display of *Longissimus thoracis* muscle of meat from Avileña-Negra Ibérica breed and significance of effect of feeding (FEED), packaging (PACK) and aging (AGING).

Longissimus thoracis											
	FEED		PACKAGING		AGING				Significance		
	CON	SIL	MAP	VAC	7 d	14 d	21 d	SEM	FEED	PACK	AGING
***n***	*72*	*72*	*36*	*36*	*48*	*48*	*48*				
**Max load (Kg)**	5.01 a	4.20 b	4.67	4.55	5.13 a	4.44 b	4.24 b	0.103	<0.001	0.537	<0.001
**Toughness (Kg/cm** ^**2**^ **)**	1.87 a	1.69 b	1.83	1.74	1.80	1.77	1.78	0.030	<0.001	0.119	0.887
**WHC thawing (%)**	4.14	3.99	4.37 a	3.76 b	4.82 a	3.54 b	3.83 b	0.139	0.188	0.014	<0.001
**WHC cooking (%)**	27.89	26.95	27.07	27.77	27.86	26.50	27.90	0.366	0.279	0.290	0.150
**WHC display (%)**	4.07	4.48	3.77 b	4.79 a	-	3.95 b	4.61 a	0.163	0.510	0.001	0.032

Means with different letters are significantly different within effect (*p* < 0.05). SEM: standard error mean. Interactions not significant.

**Table 6:** Effect of feeding (concentrate (CON) vs. 70% maize silage + 30% concentrate (SIL)) and packaging (vacuum (VAC) and modified atmosphere (MAP)) and aging (7, 14 and 21 days) on texture parameters (maximum load and toughness), water holding capacity (WHC) of thawing, cooking and display of *Longissimus thoracis* muscle of meat from Avileña-Negra Ibérica breed and significance of effect of feeding (FEED), packaging (PACK) and aging (AGING).

*Semitendinosus*											
	FEED		PACKAGING		AGING				Significance		
	CON	SIL	MAP	VAC	7 d	14 d	21 d	SEM	FEED	PACK	AGING
***n***	72	72	36	36	48	48	48				
**Max load (Kg)**	4.22	4.07	4.28 a	4.01 b	4.34 a	4.12 a,b	3.98 b	0.057	0.291	0.019	0.031
**Toughness (Kg/cm** ^**2**^ **)**	2.02	2.03	2.08	1.96	1.98	2.06	2.02	0.032	0.244	0.048	0.609
**WHC thawing (%)**	4.27	4.30	4.60 a	3.96 b	4.86 a	4.10 b	3.89 b	0.131	0.823	0.013	0.006
**WHC cooking (%)**	27.93 a	26.32 b	26.52	27.74	27.90	26.40	27.08	0.241	0.023	0.116	0.284
**WHC display (%)**	6.41	6.16	5.85 b	6.71 a	-	5.48 b	7.08 a	0.193	0.151	0.016	<0.001

Means with different letters are significantly different within effect (*p* < 0.05). SEM: standard error mean. Interactions not significant.

**Table 7:** Effect of feeding (concentrate (CON) vs. 70% maize silage + 30% concentrate (SIL)) and packaging (vacuum (VAC) and modified atmosphere (MAP)) on global, tenderness and flavour acceptability of *Longissimus thoracis* (LT) muscle of meat from Avileña-Negra Ibérica breed.

LT	FEED		PACKAGING			Significance		
	CON	SIL	MAP	VAC	SEM	FEED	PACK	FEED × PACK
***n***	*240*	*240*	*240*	*240*				
**GLOBAL**	6.21	6.50	5.94 b	6.77 a	0.079	0.044	<0.001	0.930
**TENDER**	5.95 b	6.44 a	5.67 b	6.72 a	0.091	0.004	<0.001	0.459
**FLAVOUR**	6.47	6.58	6.12 b	6.91 a	0.077	0.326	<0.001	0.619

Scale of nine points: 1 = dislike extremely; 9 = like extremely. Means with different letters (a, b) are significantly different within effect (*p* < 0.05).

**Table 8:** Effect of feeding (concentrated (CON) vs. 70% maize silage + 30% concentrate (SIL)) and packaging (vacuum (VAC) and modified atmosphere (MAP)) on global, tenderness and flavour acceptability of *Semitendinosus (ST)* muscle of meat from Avileña-Negra Ibérica breed.

ST	FEED		PACKAGING			Significance		
	CON	SIL	MAP	VAC	SEM	FEED	PACK	FEED × PACK
***n***	*240*	*240*	*240*	*240*				
**GLOBAL**	5.64	5.54	5.01 b	6.16 a	0.099	0.329	<0.001	0.610
**TENDER**	5.61	5.57	5.06 b	6.12 a	0.103	0.131	<0.001	0.697
**FLAVOUR**	5.59	5.54	4.99 b	6.13 a	0.099	0.609	<0.001	0.100

Scale of nine points: 1 = dislike extremely; 9 = like extremely. Means with different letters (a, b) are significantly different within effect (*p* < 0.05).
